# Supplementary material for: Whole-organism 3D quantitative characterization of zebrafish melanin by silver deposition micro-CT
Source: eLife. 2021 Sep 16;10:e68920. doi: 10.7554/eLife.68920 (PMC8445617; doi:10.7554/eLife.68920)
Supplement: Transparent reporting form [file elife-68920-transrepform1.pdf]

Westbrook Centre
Milton Road
Cambridge CB4 1YG, UK
P 01223 855340
W elifesciences.org
T @elife

eLife's transparent reporting form

We encourage authors to provide detailed information *within their submission* to facilitate the interpretation and replication of experiments. Authors can upload supporting documentation to indicate the use of appropriate reporting guidelines for health-related research (see EQUATOR Network), life science research (see the BioSharing Information Resource), or the ARRIVE guidelines for reporting work involving animal research. Where applicable, authors should refer to any relevant reporting standards documents in this form.

If you have any questions, please consult our Journal Policies and/or contact us: editorial@elifesciences.org.

Sample-size estimation

- You should state whether an appropriate sample size was computed when the study was being designed
- You should state the statistical method of sample size computation and any required assumptions
- If no explicit power analysis was used, you should describe how you decided what sample (replicate) size (number) to use

Please outline where this information can be found within the submission (e.g., sections or figure legends), or explain why this information doesn't apply to your submission:

> The primary goal of this study was proving the concept of 3D analysis of melanin distribution and content by micro-CT imaging of silver-stained samples and as such an explicit power analysis was not used to estimate sample size prior to scanning. Instead, the sample sizes reported in this study (n=3 for each genotype stained with silver, n=1 unstained wild-type sample) were determined at the time of scanning to optimize limited time available at the synchrotron X-ray source. Sample sizes are reported both in the text of the Results and Discussion sections and in the relevant figure and table legends.

Replicates

- You should report how often each experiment was performed
- You should include a definition of biological versus technical replication
- The data obtained should be provided and sufficient information should be provided to indicate the number of independent biological and/or technical replicates
- If you encountered any outliers, you should describe how these were handled
- Criteria for exclusion/inclusion of data should be clearly stated
- High-throughput sequence data should be uploaded before submission, with a private link for reviewers provided (these are available from both GEO and ArrayExpress)

Please outline where this information can be found within the submission (e.g., sections or figure legends), or explain why this information doesn't apply to your submission:

> Synchrotron micro-CT images were acquired sequentially at one session using the same setup and parameters. For each genotype, three biological replicates were selected at random from a single clutch of eggs at the same age, silver-stained, and imaged. Additionally, an unstained control sample from the wild-type clutch of eggs was imaged. Samples were imaged over two overlapping fields of view (head and tail) which were later registered and merged into a single 3D volume for analysis, except where clearly indicated in the Results and Discussion, figures, and tables.

eLife Sciences Publications, Ltd is a limited liability non-profit non-stock corporation incorporated in the State of Delaware, USA, with company number 5030732, and is registered in the UK with company number FC030576 and branch number BR015634 at the address Westbrook Centre, Milton Road, Cambridge CB4 1YG.

Statistical reporting

- Statistical analysis methods should be described and justified
- Raw data should be presented in figures whenever informative to do so (typically when N per group is less than 10)
- For each experiment, you should identify the statistical tests used, exact values of N, definitions of center, methods of multiple test correction, and dispersion and precision measures (e.g., mean, median, SD, SEM, confidence intervals; and, for the major substantive results, a measure of effect size (e.g., Pearson's r, Cohen's d)
- Report exact p-values wherever possible alongside the summary statistics and 95% confidence intervals. These should be reported for all key questions and not only when the p-value is less than 0.05.

Please outline where this information can be found within the submission (e.g., sections or figure legends), or explain why this information doesn't apply to your submission:

> Averages (arithmetic mean) are graphed and reported with standard deviations of the mean, and graphs include plots of all data points. As described in the Results and Discussion section, comparisons between wild-type and mutant total and regional pigment volume or content were assessed using a series of one-way ANOVAs, the results of which (including exact p-values) were summarized in Table 2. For regions compared between more than two genotypes (total melanin, body melanin, right/left RPE, eye melanin, other melanin), Tukey's post-hoc tests were used to determine which regions differed significantly from wild-type; where applicable these p-values were reported in Tables 3 and 4 and on Figure 5.

(For large datasets, or papers with a very large number of statistical tests, you may upload a single table file with tests, Ns, etc., with reference to sections in the manuscript.)

Group allocation

- Indicate how samples were allocated into experimental groups (in the case of clinical studies, please specify allocation to treatment method); if randomization was used, please also state if restricted randomization was applied
- Indicate if masking was used during group allocation, data collection and/or data analysis

Please outline where this information can be found within the submission (e.g., sections or figure legends), or explain why this information doesn't apply to your submission:

> Group allocation was based on genotype, with imaging parameters held constant. Groups (genotypes) and data collection and analysis procedures are described extensively in the Materials and Methods section. Masking and randomization were not used during allocation, collection, or analysis.

Additional data files ("source data")

- We encourage you to upload relevant additional data files, such as numerical data that are represented as a graph in a figure, or as a summary table
- Where provided, these should be in the most useful format, and they can be uploaded as "Source data" files linked to a main figure or table
- Include model definition files including the full list of parameters used
- Include code used for data analysis (e.g., R, MatLab)
- Avoid stating that data files are "available upon request"

Please indicate the figures or tables for which source data files have been provided:

Numerical data for Figure 3 and Figure 5 are summarized in Table 1 and Tables 3 and 4, respectively, with source data provided as Source Data files for each table. Source Data has also been provided for the measurements in Figure 2-figure supplement 1. Reconstructions, processed normalized reconstructions, source code, and Avizo project and source files necessary for reproducing the analyses in the manuscript are publicly available in the Dryad Digital Repository (https://doi.org/10.5061/dryad.wwpzgmsjn).

eLife Sciences Publications, Ltd is a limited liability non-profit non-stock corporation incorporated in the State of Delaware, USA, with company number 5030732, and is registered in the UK with company number FC030576 and branch number BR015634 at the address Westbrook Centre, Milton Road, Cambridge CB4 1YG.
